# Food Protein-Induced Allergic Proctocolitis: The Effect of Maternal Diet During Pregnancy and Breastfeeding in a Mediterranean Population

**DOI:** 10.3389/fnut.2022.843437

**Published:** 2022-03-30

**Authors:** Emilia Vassilopoulou, Gavriela Feketea, George N. Konstantinou, Dimitris Zekakos Xypolias, Mina Valianatou, Maria Petrodimopoulou, Vasiliki Vourga, Ioannis Tasios, Nikolaos G. Papadopoulos

**Affiliations:** ^1^Department of Nutritional Sciences and Dietetics, International Hellenic University, Thessaloniki, Greece; ^2^PhD School, “Iuliu Hatieganu” University of Medicine and Pharmacy, Cluj-Napoca, Romania; ^3^Department of Pediatrics, Pediatric Allergy Outpatient Clinic, Hospital Unit of Amaliada, General Hospital of Ilia, Amaliada, Greece; ^4^Department of Allergy and Clinical Immunology, 424 General Military Training Hospital, Thessaloniki, Greece; ^5^Allergy Department, 2nd Pediatric Clinic, National and Kapodistrian University of Athens, Athens, Greece

**Keywords:** Mediterranean Diet, fish, food protein induced allergic proctocolitis, fruit, lactation, pregnancy, cooking methods, whole wheat

## Abstract

**Background:**

The aim of the current investigation was to explore the association of food protein-induced allergic proctocolitis (FPIAP) with the maternal diet during pregnancy and breastfeeding in Greek infants.

**Methods:**

A multicenter retrospective case-control study was conducted in 6 regions in Greece, with 96 mothers of infants with and 141 mothers of infants without a history of FPIAP. Maternal dietary habits during pregnancy and breastfeeding were evaluated with the following validated questionnaires: (a) The Mediterranean Diet Score and (b) The Mediterranean Oriented Culture-Specific Semi-Quantitative Food Frequency Questionnaire.

**Results:**

FPIAP was associated with cow's milk (83.6%), egg (7.3%), wheat (6.4%), and beef (6.4%) in the maternal diet. Adherence to Mediterranean Diet was similar among the mothers. Mothers of FPIAP infants consumed more vegetables. Elastic net prediction models showed that, in this Mediterranean population, increased consumption during pregnancy and lactation of common allergens, whole grain products, homemade food, fish and shellfish, and fruits was associated with a decreased risk of FPIAP. Conversely, a high intake of vegetables, sugar and total fat, and non-stick/grilled cooking, were associated with increased risk of FPIAP, as was a high intake of salt and white flour during lactation only.

**Conclusions:**

Components of a maternal Mediterranean Diet may protect against FPIAP when traditional cooking methods are adopted and fish, fruit, and whole wheat products are consumed frequently during pregnancy and breastfeeding.

## Introduction

Food protein-induced allergic proctocolitis, a major cause of colitis in infants, is a transient benign condition. Typically, food protein-induced allergic proctocolitis (FPIAP) presents in the first months of life, as a non-immunoglobulin E (non-IgE) mediated immune response to one or more foods, with inflammation in the distal colon (1), eosinophilic infiltration of the rectal mucosa, and blood in the feces (2).

Most cases of FPIAP are in breastfed infants (60%) and are triggered by food proteins consumed by the mother, mainly cow's milk, egg, soy, and corn (3). Elimination of the offending food from the maternal diet usually results in gradual alleviation of symptoms, without discontinuation of breastfeeding (4). In formula-fed infants, the triggers are mainly cow's milk and soy; hypoallergenic or extensively hydrolyzed formulas are rarely incriminated (4–10%) (5). The disease presents at a later stage in breastfed babies and with milder histopathological lesions (6).

The reported prevalence varies, possibly due to inaccurate diagnosis, ranging widely from 0.16% in healthy children to 18–64% among infants who show fecal blood, and is higher in areas with low rates of IgE-mediated food allergy (FA), such as Greece and Brazil (7). Coexistent eczema is reported in 22% and a family history of atopic disease in 25% of cases (5).

Pregnancy is a period of immunological, metabolic, and hormonal changes, which is needed to ensure normal fetal development, the timely onset of labor, and successful delivery. Recent evidence subverts the dogma that intrauterine life is sterile (8), and exposure of the fetus to bacteria/bacterial components might be altered by maternal food variety during pregnancy, affecting allergies in infancy and childhood (9).

In breastfed infants, lactation is a critical period for immune system modulation, and maternal food variety and nutritional adequacy are currently recommended for healthy infant growth, in contrast to earlier guidelines that proposed restricted diets (10). Regarding FA, the effects of maternal diet are unclear (11), but possible benefits of the Mediterranean diet (MedDiet) warrant investigation (12). The purpose of this study was to explore the association of maternal diet during pregnancy and breastfeeding in the occurrence of FPIAP in Greece.

## Methods

### Patients

For this retrospective, observational, multicenter, case-control study, conducted between May 2018 to November 2020, mothers of breastfed infants with a history of FPIAP were recruited from 6 regions in Greece including Athens, Thessaloniki, Amaliada, Volos, Kavala, and Kozani (FPIAP group). A control population was selected of mothers of breastfed infants with no history of FPIAP, matched for age, sex, and place of residence [healthy control (HC) group]. The study was approved by the Hospital Ethics and Scientific Committee of the Amaliada General Hospital of Ilia and the Medical Association of Thessaloniki on behalf of all study centers (Study IDs: 53/2021 and 4170/2018), and conducted in accordance with the code of Ethics of the World Medical Association (Declaration of Helsinki). All participants were fully informed of the scope and procedures of the study and provided written consent.

### Clinical Data

The diagnosis of FPIAP was based on the history of scant bright red rectal bleeding with mucus in an otherwise healthy infant, which disappeared after a two-week maternal elimination diet and reoccurred after reintroduction of the culprit food. Infections and other causes of rectal bleeding, such as intussusception, volvulus, Hirschsprung's disease, and necrotizing enterocolitis, were excluded. We recorded the symptoms, the infant's age at onset and development of tolerance, physical examination findings, stool examination results, allergenic food(s) implicated in FPIAP, other allergies, and family history of atopy or asthma.

### Food Frequency Questionnaires

Information on maternal diet during pregnancy and breastfeeding was collected by a personal interview with a qualified dietician, using the following questionnaires:

a) The Mediterranean Diet Score Questionnaire, which estimates adherence to the MedDiet, recording consumption of the 11 main components (non-refined cereals, fruit, vegetables, potatoes, legumes, olive oil, fish, red meat, poultry, full-fat dairy products, and alcohol). The resultant MedDiet score is categorized: 0–13: no adherence; 14–27: insufficient; 28–41: satisfactory; 42–55: very good adherence (13).
b) The Mediterranean Oriented Culture Specific Semi-Quantitative Food Frequency Questionnaire, which includes 221 foods, subdivided into 22 food sections: (1) white grain products, including rice and potato; (2) whole wheat grain products; (3) breads/pastries; (4) stews; (5) pulses; (6) raw and cooked vegetable salads; (7) fruit and homemade juices; (8) nuts; (9) milk/dairy products; (10) meat/traditional meat dishes; (11) red meat products; (12) white meat products; (13) eggs; (14) fish/seafood; (15) fats/spreads (including olive oil); (16) traditional dips/sauces/dressings; (17) sugar/sweet preserves/confectionary; (18) “ready-to-eat” foods; (19) chips/salty puffed snacks; (20) herbals/teas; (21) soft drinks/nonalcoholic beverages; (22) alcoholic drinks. Four additional questions concern (23) addition of extra salt, (24) use of non-stick casseroles/grilled food, and (25) traditionally cooked Mediterranean homemade food; (26) food supplements.

Food consumption frequency is scored as follows: (1) never or less than once per month, (2) 1–3 times per month, (3) once per week, (4) 2–4 times per week, (5) 5–6 times per week, (6) once per day, (7) 2–3 times per day, (8) 4–5 times per day, and (9) 6 or more times per day (14).

### Statistical Analysis

The data were shown as mean (±*SD*) or median and IQR. Statistical analysis included inferential analysis, statistical tests, and modeling, using R software (version 4.04) and R studio (version 1.4.1106) (Boston, USA). The normality of the distribution of continuous variables was checked using Shapiro-Wilk's test. Comparisons between the FPIAP and HC groups were made by *t*-test (normal distribution) and Wilcoxon test (non-normal distribution). *P*-values ≤ 0.05 were considered statistically significant. To investigate the FPIAP risk in relation to the maternal diet during pregnancy and breastfeeding, the elastic net regression model was applied, which combines Lasso and Ridge regression (15), using the glmnet package in R (version 4.1-1).

## Results

The study included 96 mothers and their breastfed infants with the diagnosis of FPIAP made by a pediatric allergist (FPIAP group), and 141 mothers with healthy breastfed infants (HC group). The mean age of the FPIAP infants (48 boys, 50%) was 18 ± 12.14 months and that of the HC infants (62 boys, 44%) 18.7 ± 12.8 months. The birth weight showed no significant difference between groups. The mean current height in HC infants (86.09 ± 12.31 cm) was lower than that in FPIAP infants (93.83 ± 9.5 cm) (*p* < 0.01) (Table A in Appendix).

The median age at diagnosis and resolution of FPIAP was 2 months (IQR 1–3 months) and 12 months (IQR 11–14 months), respectively. The symptoms were the following: blood in feces (80.1%) and mucus in feces (79%); 2.7% presented anemia and 0.9% presented hypoalbuminemia. The symptoms were induced by maternal consumption of cow's milk (83.6%), egg (7.3%), wheat (6.4%), and beef (6.4%), and by lamb, peanut, rice, pear, grape, and fish (1% each) (Table B in Appendix). After diagnosis, the mothers continued breastfeeding, following an elimination diet for the offending food until FPIAP was resolved.

### Other Allergies in the Infants

Table 1 presents the allergy history of the study infants. Among the 96 infants with a history of FPIAP, at the age of data collection, 83 self-report reactions to foods including cow's milk at 59%, egg at 8%, wheat at 4%, peanut at 1%, soya at 1%, beef at 5.2%, lamb at 1%, apple at 1%, and the carrot at 1%. Wheezing was present in 8.3%, while the other 22% had eczema. In contrast, of the 141 infants in the HC group, only two had FA (one to milk and one to apple), one (0.7%) had asthma, and 2% had eczema.

**Table 1:** Allergy history of study infants, other than food protein-induced allergic proctocolitis (FPIAP).

	**All infants (*n* = 237)**	**Healthy control infants (HC) (*n* = 141)**	**Infants with FPIAP (*n* = 96)**
**Food allergy**			
Milk	58 (24%)	1 (0.7%)	57 (59%)
Egg	8 (3.4%)	0 (0%)	8 (8.3%)
Wheat	4 (1.7%)	0 (0%)	4 (4.2%)
Peanut	1 (0.4%)	0 (0%)	1 (1.0%)
Soya	1 (0.4%)	0 (0%)	1 (1.0%)
Beef	5 (2.1%)	0 (0%)	5 (5.2%)
Lamp	1 (0.4%)	0 (0%)	1 (1.0%)
Hazelnut	2 (0.8%)	0 (0%)	2 (2.1%)
Chicken	1 (0.4%)	0 (0%)	1 (1.0%)
Apple	2 (0.8%)	1 (0.7%)	1 (1.0%)
Carrot	1 (0.4%)	0 (0%)	1 (1.0%)
Sheep/goat	1 (0.4%)	0 (0%)	1 (1.0%)
**Asthma**	9 (3.8%)	1 (0.7%)	8 (8.3%)
**Eczema**	23 (9.7)	2 (1.4%)	21 (22%)

### Family Characteristics

**Figure 1:**
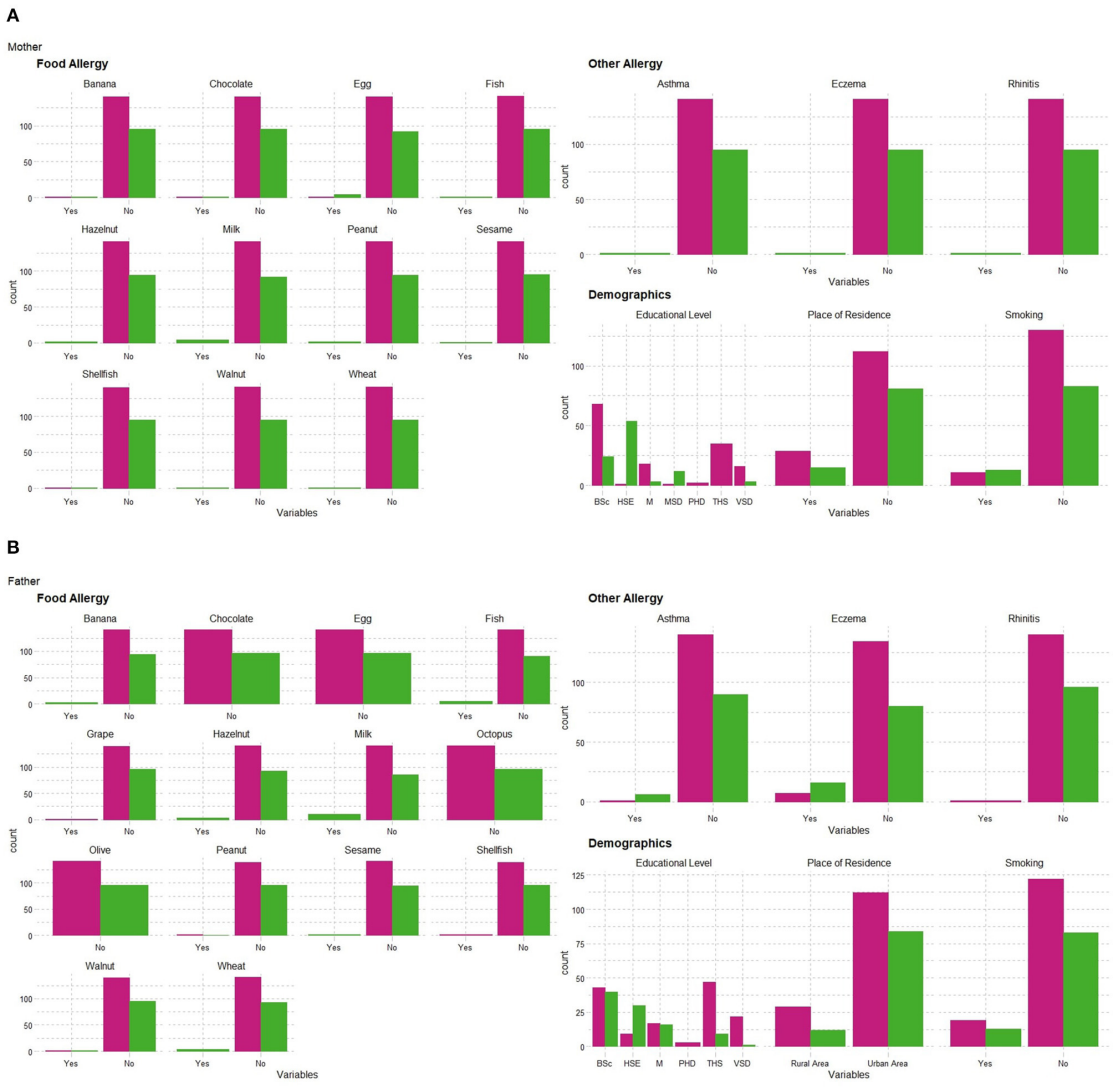
Family demographic characteristics and allergy history: **(A)** mother; **(B)** father.

The family characteristics and family history of allergies of the study infants are presented in Figure 1. The FPIAP families were more frequently urban residents (85 vs. 79%). A higher parental educational level was significantly less common in the FPIAP group (mothers 27% and fathers 24.5%) than in the HC group (mothers 62% and fathers 59%). Maternal smoking was more frequent in the FPIAP group (14 vs. 7.8%), and 13% of fathers in both groups were smokers (Figure 1A).

Infants with FPIAP more often had a family history of allergy/atopy; 20% of FPIAP mothers had documented allergy/atopy (17% FA, 1% asthma, and 1% allergic rhinitis), compared with 2.8% of HC mothers who reported FA. FPIAP fathers also had a higher rate of reported allergy/atopy, specifically FA (25%), asthma (5.4%), and eczema (14.5), than HC fathers (FA in 2.7% and 0% asthma and eczema)) (Figure 1B).

Regarding siblings, 69/96 FPIAP infants and 37/141 of the HC infants were the only children in the family. Among the infants with siblings, in the FPIAP group (27 infants with 41 siblings), a documented FA in a sibling was reported in 58.5% (12/41 milk, 7/41 egg, 3/41 fish, 4/41 shellfish, 2/41 wheat, 2/41 peanut, 2 hazelnuts, 2/41 walnut, 2/41 pepper allergy), and 15/41 had asthma, 12/41 eczema, and two (4.8%) had a history of FPIAP. Among the HC siblings (104 HC infants with 107 siblings), only 3.7% reported a FA (2/107 milk, 1/107 fish, 2/107 pepper allergy), 2/107 had a history of FPIAP, and one of eczema.

### Maternal Dietary Habits

#### Mediterranean Diet

Overall, the study mothers showed satisfactory adherence to the MedDiet, similar in the two groups (MedDiet score: FPIAP 33.87 vs. HC 33.38, *p* = 0.38). No differences in the total MedDiet score were observed among women living in the different Greek regions. No significant dietary changes were found between pregnancy and breastfeeding. Significant differences in the consumption of vegetables and olive oil were recorded between the FPIAP and the HC mothers in both study periods: for vegetables 2.19 ± 0.91 vs. 1.85 ± 0.99 in pregnancy, *p* = 0.008, and during breastfeeding 2.1 ± 0.89 vs. 1.8 ± 0.95, *p* = scores of 0.018; for olive oil 4.78 ± 0.64 vs. 4.56 ± 0.79 in pregnancy, *p* = 0.023, and during breastfeeding 4.35 ± 1.09 vs. 4.74 ± 0.74, *p* = 0.003.

#### Food Frequency Consumption During Pregnancy

According to their responses on the Mediterranean Oriented Culture-Specific Semi-Quantitative Food Frequency Questionnaire, maternal consumption of certain food groups differed significantly between groups, as well as between pregnancy and lactation, as shown in Table 2.

**Table 2:** Food consumption and food supplement use during pregnancy and breastfeeding of mothers of infants with FPIAP and healthy control subjects (HC).

	**FPIAP**	**HC**	***p*-value**
**Pregnancy**			
Fish and shellfish	1 (±0.7)	1.2 (±0.6)	0.003
Vegetables	1.9 (±0.9)	1.6 (±0.5)	0.02
Folic acid	1.4 (1)	1.5 (1)	0.01
n-3	2.21 (±1.22)	2.1 (±0.93)	0.04
Multivitamin use	1.7 (1)	1.9 (1)	<0.01
Sweets	1.3(±0.7)	1.1(±0.6)	0.06
Whole wheat products	0.9 (±0.7)	1.1(±0.6)	0.07
**Breastfeeding**			
Vegetables	2.3 (±1.9)	1.8 (±1.6)	0.05

During pregnancy, FPIAP mothers consumed less fish and shellfish (*p* = 0.003) and more vegetables (*p* = 0.02), than HC mothers, and they used less food supplements, specifically folic acid (*p* = 0.01), multivitamins (*p* < 0.01), and omega-3 (*p* = 0.04).

During breastfeeding the FPIAP mothers consumed significantly more vegetables than during pregnancy (*p* = 0.05), while vegetables' consumption was not altered by the HC mothers in the two study periods (*p* > 0.05).

#### Prediction Models of FPIAP Risk According to Maternal Diet During Pregnancy and Breastfeeding

**Figure 2:**
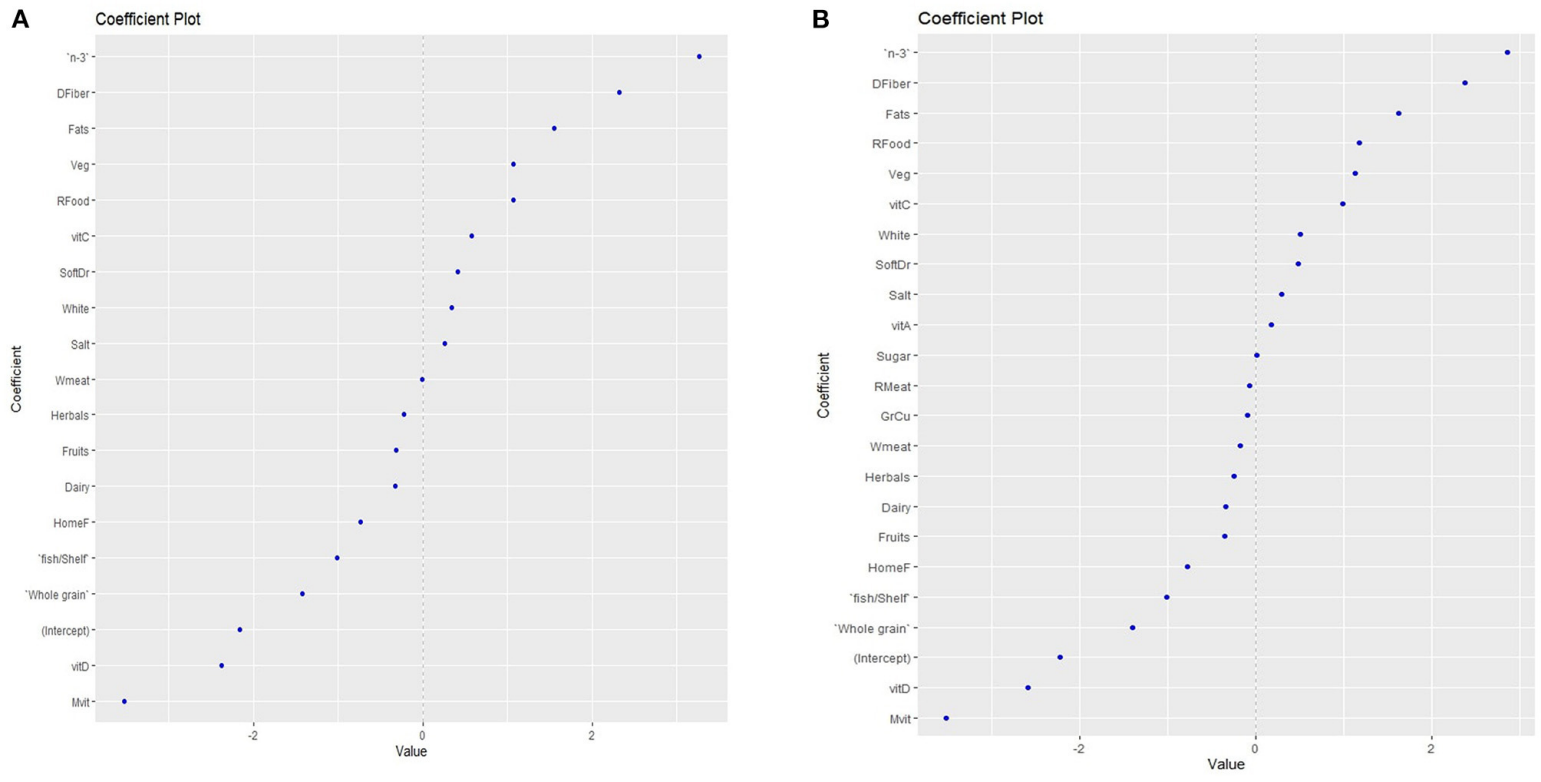
Maternal dietary factors that are associated with increased food protein-induced allergic proctocolitis (FPIAP) risk or protection, as predicted with the Elastic Net Regression model during pregnancy. Food products with values < 0 protect, while values > 0 increase the risk, **(A)** during pregnancy **(B)** during breastfeeding. n-3 = omega 3 polyunsaturated fatty acids; DFiber = dietary fiber; Veg = vegetables; RFood = ready made food; vitC = vitamin C; SoftDr = soft drinks; White = white grains; White meat = white meat/meat products homemade food; fish/Shelf = fish/ shellfish; Rmeat = red meat/meat products; Mvit = multivitamins.

Elastic net regression was used to construct multifactorial models to explore the effect of maternal dietary factors on FPIAP risk. The pregnancy model presented high statistical significance (*p* < 0.01) and good accuracy (accuracy = 0.83), with an area under the curve (AUC) of 0.87, confirming the ability of the model to distinguish maternal dietary factors affecting FPIAP. According to this model, pregnancy factors associated with FPIAP diagnosis were n-3 and vitamin C supplements, sugary products, vegetables, total fat, and the use of non-stick kitchenware and grills. Protective factors were traditionally cooked homemade food, dairy products, nuts, whole grain products, fish and shellfish, fruits, multivitamins, vitamin A, vitamin D, B-complex and folic acid, and dietary fiber (Figure 2A).

The breastfeeding model did not reveal statistical significance (*p* = 0.08). However, there was a trend towards a non significant association of FPIAP cases with increased consumption of dietary fiber, total fat, vegetables, sugar/sugary products and soft drinks, white flour products, and salt, whereas no significant protective factors included increased intake of fruits, fish and shellfish products, whole grain products, homemade food, vitamin D, and multivitamin supplementation (Figure 2B).

## Discussion

This retrospective, observational, multicenter case-control study, the first to explore the risk of FPIAP in relation to maternal dietary habits during pregnancy and lactation, provided evidence that the traditional MedDiet can be protective against FPIAP, when whole wheat products, fish, and fruit are consumed frequently by the mother, using traditional cooking methods. A high intake of fat, sugar, and vegetables increases the risk of FPIAP, together with the use of grills and non-stick kitchenware. As in other allergies, the inclusion of common food allergens in the maternal diet appears to protect infants from FPIAP.

Current evidence does not support maternal dietary restrictions during pregnancy or lactation for prevention of allergic disease in the offspring (10, 16); consumption of allergenic foods in pregnancy (17) and during lactation is reported to prevent allergic sensitization to these foods (17, 18), and in line with this, frequent consumption of common allergenic foods (dairy products, nuts, whole-grain foods, and fish/shellfish) as part of a balanced diet, was associated with a reduction in the risk of FPIAP (17, 19, 20).

Earlier studies suggest that the MedDiet is associated with reduced allergic outcomes (21), and a systematic review confirmed the beneficial effect of the MedDiet during pregnancy and lactation (12). In the current study, both groups of mothers recorded similar satisfactory adherence to the MedDiet, thus allowing the study of the impact of specific foods within this dietary pattern. Home-cooked food, exemplifying Mediterranean dietary habits, appeared to be associated with a protective effect. The apparent beneficial effect of specific components of MedDiet and/or traditional cooking methods in the protection against allergies has generated interest in a proposal for a large prospective randomized controlled trial (RCT) during pregnancy to assess its effect on infant allergy prevention (22, 23).

Conversely, a high-fat diet and the use of non-stick kitchenware and grills were associated with FPIAP. The consumption of olive oil did not appear as a significant individual factor as it did not differ between our study groups, but the total fat intake was a risk factor for FPIAP. Results from a mouse model suggest that a high-fat diet increases the risk of FA due to microbiome alterations (24). Grilling food, mainly meat, at home may affect the fetal immune system, and probably aggravate the risk of food allergies in neonates and children, via the carcinogenic compounds produced at high temperatures (25).

Sugary products and soft drinks were associated with an increase in the risk of FPIAP in our population, in agreement with a recent metanalysis of high sugar consumption during pregnancy and lactation and allergies in the offspring (26). Moreover, high salt intake during lactation appeared to increase the risk of FPIAP. Documentation is lacking about the effect of salt intake during pregnancy and lactation on infant allergy risk.

Whole grain products were associated with a reduced risk of FPIAP in both periods, but white flour consumption during breastfeeding was associated with an increase in the risk of FPIAP. Dietary fiber supplementation during pregnancy appeared to exert a beneficial effect. Dietary fiber acts as a prebiotic, ensuring gut microbial biodiversity during pregnancy. The microbiome is transferred from mother to offspring. Loss of microbial communities or species occurs in the mother-infant dyad when the diet is rich in highly processed foods (27). Breast milk benefits the infant gut microbiome diversity, and formula-fed infants lack dominant microbes; formula enriched with symbiotics have not increased gut biodiversity or provided protection in high-risk infants (28–30). Increased insoluble dietary fiber intake during pregnancy is associated specifically with reduced risk of wheeze and increased risk of eczema in the infant (31). Conversely, maternal fiber supplementation during lactation was associated with an increase in the risk of FPIAP.

An unexpected finding was the link of vegetable intake with FPIAP. Other studies associated maternal vegetable intake with allergy prevention (25, 32), although offspring of women with a higher intake of total vegetables, folate-rich, and green and yellow vegetables were reported to have a higher risk of allergies, including FA (33). In countries where the MedDiet is widespread, vegetables are frequently incriminated in FA (34–36). In Greece, pregnant and lactating women are encouraged to consume washed and peeled fruits without restrictions, but they are often advised to consume only specific vegetables during the following events: a) during pregnancy, to eat only cooked or boiled vegetables, for prevention of toxoplasmosis, transmitted through vegetables grown in soil contaminated with cat feces (37); b) during lactation, to prefer green leafy vegetables, but to avoid vegetables that may cause colic in the baby, such as broccoli (38). Cooking methods affect the antioxidant vitamins, with loss in vitamins C, E, β-carotene, and K (39), dietary fiber, and slight increases in insoluble dietary fiber (40).

A high intake of seasonal fruits during pregnancy and lactation, both fresh and dried, was associated with a reduced risk of FPIAP. The seasonal availability of a wide variety of Mediterranean fruits rich in antioxidants protects against oxidative exposures, abating allergy risk during pregnancy, lactation (12) infancy (41) and childhood (42, 43). The high fruit content of soluble dietary fibers enhances the development of a healthy intestinal microbiota (23), and fruit antioxidants, including polyphenols, carotenoids, and vitamins A and C, are anti-inflammatory (44). Vitamin C supplements increased the risk of FPIAP, and relevant data are conflicting, indicating low to no protection against allergies (21).

Several researchers report a protective effect of high fish and seafood intake in pregnancy and infancy against allergic diseases in childhood (45–47), and our study confirmed a reduced risk of FPIAP. In our population, n-3 long-chain polyunsaturated fatty acid (LC-PUFA) supplementation was a risk factor for FPIAP, and although n-3 PUFA supplementation has been suggested for allergy prevention (48), especially in lower socioeconomic populations (49) its benefits are obscure (50–52).

Multivitamin supplements, folic acid, vitamins A, D, and B-complex during pregnancy and vitamin D and multivitamin supplements during lactation were associated with a reduced risk of FPIAP, and other researchers have reported vitamin protection against allergy outcomes in the offspring, mainly in undernourished mothers, especially from vitamin A and folic acid, and also iron (53, 54).

Vitamin D supplementation, in both pregnancy and lactation, appeared to reduce the risk of FPIAP, and other researchers report that it protects against asthma and FA in the offspring (55, 56). Inadequate levels of vitamin D in infants have been associated with clinical manifestations of cow's milk allergy, including non-IgE forms (57), and the role of vitamin D in the prevention of FA (58) and other atopic disease is documented (55, 56, 59).

Food protein-induced allergic proctocolitis (FPIAP) is frequently, but not exclusively, caused by cow's milk protein, even in exclusively breastfed infants (3, 60, 61). In 83.6% of the cases proved by the challenge in this study, cow's milk was incriminated in FPIAP.

A striking finding in our study was that approximately 25% of our FPIAP infants had parents with higher education compared with 60% of HC infants. In the Mediterranean area, parental tertiary education has been found associated with high consumption of fruits and vegetables, and low childhood asthma prevalence (62).

In line with other reports (63), the FPIAP infants more often had a family history of allergy and atopy than the HC infants. Infants with FPIAP are reported to develop IgE-mediated FA (64).

Children who are pre- and postnatally exposed to tobacco smoke have a significantly higher risk of FA (65), and in our study maternal smoking was associated with FPIAP. Paternal smoking was equivalent in the two groups and had less effect, probably because fathers spend less time in close contact with the infants (66).

Our results support the need for increased counseling on maternal diet during pregnancy and breastfeeding, especially when there is a family history of allergy, in the context of the prevailing Mediterranean diet, as certain foods, nutrients, and cooking methods appear to be associated with FPIAP.

A limitation of the current investigation was the retrospective design, data based on self-reported questionnaires, and recall bias when they report their past eating habits during pregnancy and lactation. There is a need for a prospective, intervention study to investigate the apparent benefits of the specific components of the Mediterranean diet model for infant allergy prevention during pregnancy and lactation.

## Conclusion

In this first cross-sectional, multicenter study on the association of maternal diet during pregnancy and breastfeeding with the development of FPIAP in the offspring, we found that frequent consumption of common allergens, food home-cooked according to the traditional Mediterranean customs, wholewheat grains, fruits, fish and shellfish, and folic acid, vitamins D, A, B-complex, and multivitamin supplementation appeared to protect against FPIAP in infancy. Conversely, a diet with high content of fat, sugary products, salt, vegetables and dietary fiber, vitamin C and n-3 PUFA supplementation, and use of non-stick kitchenware/grills appeared to increase the risk of FPIAP. A family history of allergy was a strong predisposing factor. These results show that elements of the MedDiet may be protective. This evidence should be further evaluated in prospective intervention studies and used to develop dietary guidelines for mothers during pregnancy and lactation, for the prevention of FPIAP.

## Data Availability Statement

The raw data supporting the conclusions of this article will be made available by the authors, without undue reservation.

## Ethics Statement

The studies involving human participants were reviewed and approved by Hospital Ethics and Scientific Committee of the Amaliada General Hospital of Ilia (53/03-09-2021) and the Medical Association of Thessaloniki (4170/18-4-2018). Written informed consent to participate in this study was provided by the participants' legal guardian/next of kin.

## Author Contributions

EV and NP: conceptualization. EV, GF, GK, MV, MP, and IT: data collection. EV and GF: drafting the article. EV and DZ: data analysis and interpretation. All authors: critical revision and approval.

## Conflict of Interest

The authors declare that the research was conducted in the absence of any commercial or financial relationships that could be construed as a potential conflict of interest.

## Publisher's Note

All claims expressed in this article are solely those of the authors and do not necessarily represent those of their affiliated organizations, or those of the publisher, the editors and the reviewers. Any product that may be evaluated in this article, or claim that may be made by its manufacturer, is not guaranteed or endorsed by the publisher.

## Abbreviations

MedDiet: Mediterranean diet
FPIAP: Food Protein-Induced Allergic Proctocolitis
FA: Food Allergy
HC: Healthy Control.

## Appendix

**Table A:** Characteristics of infants with food protein-induced allergic proctocolitis (FPIAP) and healthy control subjects (HC) included in the study.

	**FPIAP (*n* = 96)**	**HC (*n* = 141)**	***P-*value**
Age (months)	18.67 ± 12.11	18.01 ± 12.83	0.68
Birth weight (kg)	3.2 ± 0.42	3.15 ± 0.41	0.06
Birth length (cm)	50.36 ± 3.17	50.66 ± 2.03	0.40
Current weight (kg)	13.56 ± 2.68	13.73 ± 6.77	0.79
Current length (cm)	93.83 ± 9.5	86.09 ± 12.31	<0.01
Sex	48M/48F	62M/79F	

**Table B:** Sympstoms of FPIAP and foods implicated (*N* = 96).

**Symptoms**	
Blood in stools	89 (93%)
Mucus in stools	87 (91%)
Anemia	3 (3.1%)
hypoalbuminemia	1 (1%)
**Foods implicated in FPIAP**	
Cow's milk	92
Egg	8
Wheat	6
Beef	7
Soya	3
Corn	2
Peanut	1
Hazelnut	1
grape	1
Rice	1
Fish	1
Lamb	1
